# Screening plant growth-promoting bacteria from the rhizosphere of invasive weed *Ageratina adenophora* for crop growth

**DOI:** 10.7717/peerj.15064

**Published:** 2023-03-10

**Authors:** Yun Xia, Hongbo Zhang, Yu Zhang, Yuyu Zhang, Jiani Liu, Robert Seviour, Yunhong Kong

**Affiliations:** 1 Yunnan Urban Agricultural Engineering & Technological Research Centre, Kunming University, Kunming, Yunnan, China; 2 School of Agriculture and Biotechnology, Kunming University, Kunming, Yunnan, China; 3 Microbiology Department, La Trobe University, Bundoora, Victoria, Australia; 4 Kunming Key laboratory of Hydro-ecology Restoration of Dianchi Lake, Kunming University, Kunming, Yunnan, China

**Keywords:** Plant growth-promoting rhizobacteria, *Ageratina adenophora*, N-fixation, Indole-3-acetic acid production, Amino-cyclopropane-1-carboxylate deaminase production, Greenhouse pot experiment

## Abstract

Plant-growth promoting rhizobacteria (PGPR) play a vital role in soil fertility and crop production. The rhizosphere of many crop plants has been well documented by screening PGPR for their plant-growth promoting (PGP) mechanisms. However, the rhizosphere of grass species that may act as potential habitats for novel PGPR remains relatively unexplored. *Ageratina adenophora* is a noxious weed that has invaded more than 40 tropical and subtropical countries in Asia, Oceania, Africa, and Europe. Its presence has led to changes in plant species composition, reducing their biodiversity and destroying ecosystem function. In this study, we screened 1,200 bacterial strains isolated from the rhizosphere soil of *A. adenophora* in three floristic regions in Yunnan Province, China. Samples were screened for their *in vitro* ability for N-fixation, production of the plant growth regulator indole-3-acetic acid (IAA), and the synthesis of 1-amino-cyclopropane-1-carboxylate (ACC) deaminase, which controls the levels of ethylene in developing plant roots. We found that 144 strains showed at least one of these PGP attributes. 16S rRNA gene sequencing showed that most (62.5%) of the samples were bacteria closely related to members of the genera *Pseudomonas* (27 strains), *Providencia* (20 strains), *Chryseobacterium* (14 strains), *Ensifer* (12 strains), *Enterobacter* (nine strains), and *Hafnia* (eight strains). Their abundance and biodiversity in the soil of individual floristic regions correlate positively with the invasion history of *A. adenophora*. From these PGP bacterial strains, KM_A34 (*Pantoea agglomerans*), KM_C04 (*Enterobacter asburiae*), and KM_A57 (*Pseudomonas putida*), which had the greatest *in vitro* ability of N-fixation, and IAA and ACC deaminase production, respectively, were selected. The strains were evaluated for their effect on the seed germination and growth of soybean, faba bean, pea, wheat, and Chinese cabbage other than *A. adenophora*. Chamber experiments showed these strains significantly (*P* < 0.05) increased (14.2–43.4% over the controls) germination rates of the soybean, faba bean, pea, and/or Chinese cabbage seeds. They also reduced relative seed germination times (20.8–48.8% over the controls) of soy bean, faba bean and/or wheat seeds. Greenhouse pot experiments showed that they significantly (*P* < 0.05) promoted the aboveground and belowground height of plant foliage (12.1–23.1% and 11.5–31.4% over the controls, respectively) and/or the dry weights (16.1–33.5% and 10.6–23.4% over the controls, respectively) of the soy bean, faba bean, pea, wheat and/or Chinese cabbage. These data indicate that the rhizosphere microbiota of *A. adenophora* contain a PGPR pool that may be used as bioinoculants to improve the growth and productivity of these crops.

## Introduction

Plants develop mutualistic relationships with soil microbes during their growth and development (Gouda et al., 2018). It is estimated that they release 20–50% of their photosynthetically-obtained carbon into the soil rhizosphere (Kuzyakov & Domanski, 2000). Soil microbes utilize these exudates, together with sloughed-off root cells, as their source of nutrients (Dennis, Miller & Hirsch, 2010) and, in turn, beneficially impact the host plant. These organisms include the plant growth promoting rhizobacteria (PGPR), which encourage plant growth by N-fixation and the production of plant growth regulators, including indole-3-acetic acid (IAA) and siderophores, which supply plants with required amounts of iron (Glick et al., 1999). These rhizobacteria synthesize cyanide for antagonistic activity against phytopathogenic soil microbes, together with 1-amino-cyclopropane-1-carboxylate (ACC) deaminase, which controls the levels of ethylene in developing plant roots (Bhattacharyya & Jha, 2012). PGPR may also be responsible for the solubilization of phosphate and potassium, the degradation of environmental pollutants, and the production of antibiotics and/or lytic enzymes that suppress plant pathogens, as well as heavy metal detoxification and salinity tolerance (Gouda et al., 2018). PGPR activities also play a vital role in soil fertility (Mahmood et al., 2016). Their interactions with plants have been exploited commercially and their potential importance to sustainable agriculture has generated more interest in their research (Gupta et al., 2015).

The rhizosphere microbiomes of many crop plants have been well documented in terms of screening PGPR for their plant-growth promoting (PGP) mechanisms (Bhattacharyya & Jha, 2012; Gouda et al., 2018; Gupta et al., 2015). However, grass species representing potential habitats for novel PGPR remain relatively unexplored (Baber et al., 2018; Sarathambal et al., 2014). *A. adenophora* is a notorious weed originating in Mexico and Costa Rica, which has now invaded more than 40 tropical and subtropical countries in Asia, Oceania, Africa, and Europe (Poudel et al., 2019), leading to changes in plant species composition in the invaded areas, reducing their biodiversity, and destroying ecosystem function (Devine & Fei, 2011; Fei, Phillips & Shouse, 2014; Walsh, Carpenter & Vander Zanden, 2016). Its invasion has changed the composition of the rhizosphere bacterial community in affected soils (Kong et al., 2017; Xia et al., 2021; Zhao et al., 2019), enriching free N-fixation bacteria (Xu et al., 2012) and arbuscular mycorrhizal fungi (Sun, Gao & Guo, 2013; Xia et al., 2021), and altering soil nutrient availability to facilitate their growth and competitiveness (Niu et al., 2007; Zhao et al., 2019). All such attributes make it an excellent model for seeking novel PGPR that are beneficial for crop growth.

In this study, we chose three sites invaded by *A. adenophora*, each in a different floristic region in the Yunnan Province of China. We isolated 1,200 bacterial strains from the rhizosphere soils of *A. adenophora*, and investigated their abilities to fix nitrogen, and produce IAA and ACC deaminase. We then selected those with the most impressive *in vitro* PGP traits and evaluated their effects on the behavior of five different crop plants as detailed below.

## Materials and Methods

### Site description and sampling strategy

**Figure 1:**
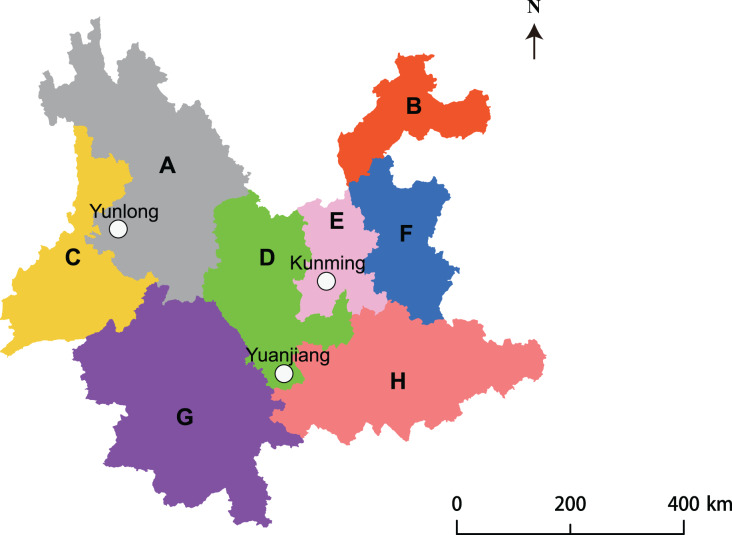
Site map showing the three study sites located at different floristic areas in Yunnan Province, China.

Three sites where *A. adenophora* invasion had occurred in different floristic regions (Li et al., 2015) of Yunnan Province, China, were chosen for study (Fig. 1). Their floristic, climatic, and soil characteristics are listed in Table 1. The site at Kunming (ca. 50 × 50 m^2^) was located 50 m from a conifer-broadleaf forest. The site at Yuanjiang (ca. 50 × 30 m^2^) was located 30 m away from a conifer-broadleaf forest, where *A. adenophora* grew sporadically among a range of native plant species. The site at Yunlong (ca. 60 × 30 m^2^) was located in a valley where *A. adenophora* grew in large patches separated by several different native shrubs and grasses.

**Table 1:** Geographic and climatic features of study sites.

Sample site	Longitude and latitude	Altitude	Invasion history of *A. adenophora*	Floristic type	Vegetation type	Climatic type	Annual average temperature	Annual average precipitation	Soil type
Kunming	24°57′N102°37′E	2,327 m	10–12 year	V	Conifer-broadleaf forest	South subtropical climate	16.5 °C	1,450 mm	Red soil
Yuanjiang	23°33′N102°03′E	1,157 m	2–3 year	IV	Conifer-broadleaf forest	Middle subtropical climate	23.8 °C	800 mm	Dry-red soil
Yunlong	25°53′N99°22′E	1,683 m	6–8 year	I	Shrubs and grasses	North subtropical climate	16.2 °C	780 mm	Yellow-brown soil

Three soil samples were collected from the rhizospheres of *A. adenophora* at each study site. Each individual sample site was separated from its neighbor by at least 2 m and all were located at the same altitude. To collect rhizosphere soil samples, individual plants were pulled up and shaken to remove the soil loosely attached to the roots, leaving only firmly attached soil, which was then used in the experiments described here. Individual soil samples were placed into sterile Whirl-Pak sample bags (Nasco, Fort Atkinson, WI, USA) and transported to the laboratory within 5 h of collection. Approximately 500 g of each soil sample was homogenized (medium treatment for 1 min) in a Waring heavy-duty blender (Lab-Biogen, Kunming, China), which was carefully cleaned after each use with 70% ethanol and passed through a 2 mm sieve before isolation of bacterial strains exhibiting PGP traits. Individual soil samples were collected from each study site on the same day between 15 August and 5 September, 2019.

### Isolation and characterization of bacterial strains with *in vitro* PGP abilities

A total of 10 g soil from each soil sample was shaken (200 rpm) in a 500 mL flask with 90 mL sterile distilled water and glass beads for 10 min to isolate and characterize bacterial strains with *in vitro* PGP abilities (PGP bacteria). The flask was left at room temperature for 30 s before the suspensions were serially diluted in a 10× dilution series with sterile distilled water. The suspensions were then used to isolate bacterial strains on agar plates containing King’s B, Luria-Bertani (LB), Lowenstein-Jensen, R2A, and Gauze’s synthetic medium no.1 agar media to maximize the isolation of different PGPR strains. A total of 400 colonies from the three soil samples from each study site, based on different colony morphologies, were subcultured onto LB medium and screened for their abilities to fix nitrogen and produce IAA and ACC deaminase, as described below.

#### Characterization of N-fixation bacteria

Isolates were screened using Ashby agar plates (Ashby, 1907) for N-fixation ability. Incubation was carried out at 28 °C for 72 h. Bacterial strains able to grow on Ashby agar plates after three subcultures were considered to have N-fixation ability. To quantify their N-fixation ability, RNA from fresh Ashby liquid medium-grown cultures (10 mL) of these strains was extracted and purified with a PureLink® RNA Mini Kit (Thermo Fisher Scientific, Shanghai, China). After quality evaluation by 1.2% agarose gel electrophoresis, a 1.0 μg aliquot of total RNA was used for reverse transcription to generate a cDNA according to the manufacturer’s instructions (SuperScript IV First-Strand Synyhesis System; Thermo Fisher Scientific, Shanghai, China). The quantification of *nifH* gene expression was performed using real-time qPCR as described by Stenegren et al. (2018) with modifications. Briefly, the following two primer pairs were used for cDNA amplification: 19F and 278R for the *nifH* gene, and 530F and 1100R for the 16S rRNA gene. The latter primer pair acted as endogenous controls, and both sets of primers were used at 10 µmol. Real-time qPCR reactions were performed in a total volume of 20 µL using the 2×SYBR MasterMix (Shangon Biotech, Shanghai, China) in an Applied Biosystems QuantStudio 3 (Thermo Fisher Scientific, Shanghai, China). DNA amplification was performed with an initial denaturation cycle at 95 °C for 10 min followed by 45 amplification cycles of 95 °C for 10 s, 60 °C for 20 s, and 72 °C for 15 s, with a final extension at 72 °C for 3 min. Melting curve analysis of the PCR products was conducted following each assay to confirm that the fluorescence signal was derived from specific PCR products and not from primer-dimers or other artifacts. Each qPCR analysis was performed in three replicates. The relative *nifH* gene expression level was calculated using the following formula: *nifH_*level = 2^−∆∆CT^, where CT is cycle threshold, ∆∆CT = (CT_nifH − CT_endogenous control) − (CT_control − CT_endogenous control). The strain KM_G25, with the lowest value of CT_nifH − CT_ endogenous control, was used as the control.

#### Characterization of IAA-producing bacteria

IAA production by these isolates was determined using a modified method from Abbas-Zadeh et al. (2010). Briefly, cultures were grown in liquid LB medium supplemented with L-tryptophan (0.5 g/L) and shaken for 72 h at 30 °C. Then, 1 ml aliquot of each was centrifuged at 8,000 rpm for 2 min, and 200 µl supernatant was mixed with the same volume of Salkowski reagent before being incubated in the dark for 30 min. IAA production was noted to be positive after the development of a pink color. Quantification was based on A_535_ values (Patten & Glick, 2002), where IAA levels (mg L^−1^) were calculated using a standard curve prepared with IAA (Sigma, Shanghai, China).

#### Characterization of ACC deaminase-producing bacteria

ACC-deaminase production was determined according to the method of Amico, Cavalca & Andreoni (2005) with modifications. Cultures were grown on DF medium for 48 h at 30 °C where (NH_4_)_2_SO_4_ was replaced with 3.0 mmol/L ACC. The incubation period was repeated three times to confirm the utilization of ACC as the sole N source. ACC-deaminase production was quantified by measuring the levels (U mg^−1^) of α-ketobutyrate produced after ACC deaminase cleavage of ACC, according to the method of Honma & Shimomura (1978).

### Identification of PGP bacteria by 16S rRNA sequencing

Genomic DNA was isolated from bacterial isolates using the Lysis Buffer for Microorganism to Direct PCR (Takara, Shanghai, China) according the protocol provided. 16S rRNA genes were amplified using universal primers 27f (5′-AGA GTT TGA TCM TGG CTC AG-3′) and 1492r (5′-CGG TTA CCT TGT TAC GAC TT-3′) (Lane, 1991) with 2×Taq MasterMix (Sangon Biotech, Shanghai, China). 16S rRNA gene amplicons were sequenced from both ends with an Applied Biosystems 3710X1 DNA Sequencer (Thermo Fisher Scientific, Shanghai, China). The nucleotide sequences obtained were aligned into consensus sequences using Mega11 software (Kumar, Tamura & Nei, 1993) and were then analyzed using BLASTn (NCBI) to identify their nearest neighbors. Distance trees showing all PGP bacterial strains were built with the Mega11 software.

### Chamber germination experiments

The three PGP bacterial strains with the greatest abilities for N fixation, IAA and ACC deaminase production, respectively (KM_A34, KM_C04, and KM_A57) (Table S1) were selected and their PGP effects on the germination of soybean (*Giycine max* L. Merrill), faba bean (*Vicia faba* L.), wheat (*Triticum aestivum* L.), pea (*Pisum sativum* L.), Chinese cabbage (*Brassica campestris* L.), and *A. adenophora* were examined. Seeds of these crop plants were purchased from a local seed market. *A. adenophora* seeds were collected from *A. adenophora* plants from the Kunming site in May 2019.

Chamber experiments were designed with complete randomization, with six replicates per treatment. They included a set of non-inoculated controls set up according to the procedures described by Amogou et al. (2018) with slight modifications. Briefly, seeds were disinfected by soaking for 2 min in a sodium hypochlorite solution (0.024%) and then rinsed repeatedly with sterile distilled water and vortex agitation. For bacterial inoculations, PGP strains were grown in LB broth for 48 h at 28 °C. Cells were collected by centrifugation (12,000 g, 10 min), washed twice with 0.9% NaCl sterile solution, and adjusted to 10^9^ CFU mL^−1^. The seeds were added to suspensions of the selected bacterial strain. After inoculation, 12 seeds of each were dispersed equidistantly on a paper towel previously moistened with 10 ml of sterile distilled water and were deposited in sterile petri dishes of 11.8 cm diameter. Plant seeds were covered by another paper towel dampened with 10 ml water and the petri dishes were incubated at 30 °C in a growth chamber in the dark. The numbers of germinated seeds of each were counted to determine their germination percentages. The germination time (GT, number of days post planting) was calculated using the following formula: GT = ∑(Gi × i)/∑Gi, where i is the number of days between seed sowing (day 0) and seed germination; and Gi is the number of seeds germinated on day i (Zhang et al., 2014).

### Green house pot experiments

The effects of the three PGP bacterial strains on soybean, faba bean, wheat, pea, Chinese cabbage, and *A. adenophora* growth were also determined. The soil used for the potted trials was taken from the campus garden of Kunming University. The soil was air dried initially for 2 weeks and sieved to a particle size of <2 mm. The physicochemical properties of the soil were as follows: pH 7.06; total organic carbon 45.46 g kg^−1^; total nitrogen 4.53 g kg^−1^; available P 16.89 mg kg^−1^; and available potassium 130.39 mg kg^−1^. Polyvinyl chloride (PVC) pots that measured 15 cm in diameter and 40 cm in height were used. Each pot was filled with 8 kg of soil and watered to 2/9^th^ of their maximum holding capacity 24 h prior to sowing.

Three holes were made in the soil surface in each pot and one seed that had been inoculated with bacteria was gently planted in each hole. The holes were then filled. Pot experiments were conducted in a greenhouse (altitude 1,890 m; 24°58′N; 102°48′E) at Kunming University from March to August 2020. The temperature in the study area varied between 18.3 °C and 28.8 °C. On the 15^th^ day after seed germination, the least vigorous of the plants were removed from their respective planters. A root dip inoculation with 10 ml of each suspension of the PGP bacterial strains containing approximately 10^9^ cells per mL was performed. Pots were watered at 1/9^th^ of their maximum water retention capacity every 48 h after seed germination until the end of the experiments. Six replicate experiments (pots) were used for each treatment. The same number of replicates with uninoculated seeds were performed as controls. Root dips were performed with 10 mL sterilized water on the 15^th^ day after seed germination. Data, including aboveground and belowground heights and dry weights, were collected 60 days after seed planting. The data were analyzed for each plant. In order to determine the dry weight, the plant components were placed into a hot air oven at 60 °C until a dry weight was established.

### Physicochemical analysis of soil samples

Physiochemical analyses of soil samples were carried out following procedures described by Kong et al. (2017). Briefly, soil organic matter composition was determined using the K_2_Cr_2_O_7_-H_2_SO_4_ oxidation method (Nelson & Sommers, 1996). Total nitrogen levels were measured using the Kjeldahl method (ISO 11261, 1995). The available phosphorus and potassium levels were estimated using methods described by Kuo (1996) and Helmke & Sparks (1996), respectively. The pH (1:2.5 solution of soil to water) was measured using a pH meter (Mettler-Toledo International Inc., Shanghai, China).

### Statistical analysis

Mean values and the least square difference of the relative *nifH* gene expression level, the IAA and ACC deaminase concentrations, the relative germination rate (GR) and GT, the relative aboveground and belowground heights, and the dry weights were calculated using SPSS (version 16.0; SPSS, Inc., Chicago, IL, USA). The results were expressed as mean ± standard error for each treatment. Relative GR, GT, aboveground and belowground heights, and dry weights were calculated as percentages of the values of individual germination and growth parameters measured for each individual plants in the treatment groups divided by the value measured for its plants in the control groups. Significant differences in these parameters between the treatment groups and their control groups were determined using the Kruskal–Wallis test with SPSS (version 16.0). Significant differences were established at *P* < 0.05. The correlation between the invasion years of *A. adenophora* to the genus and strain number of its rhizosphere PGP bacteria was analyzed using corrplot in RStudio (RStudio Team, 2017).

## Results

### Isolation of bacteria from the rhizosphere soils of weed *A. adenophora*

To ensure a comprehensive screening of PGPR from the rhizosphere soil of the weed *A. adenophora*, 1,200 bacterial stains were isolated from the three rhizosphere soil samples (400 strains from each soil sample) belonging to *A. adenophora* located in three floristic areas (Fig. 1). The sample areas included Kunming with a south subtropical climate, Yuanjian with a middle subtropical climate, and Yunlong with a north subtropical climate in Yunnan, China (Table 1). The study site at Kunming had the longest *A. adenophora* invasion history (10–12 years) followed by that at Yunlong (6–8 years) and Yuanjiang (2–3 years). Each site had a different soil type (Table 1); the soil from *A. adenophora* in Kunming contained a higher (*P* < 0.05) level of organic C and lower level of NO_3_^−^-N than those from Yuanjian and Yunlong. The soil from Yunlong had a higher (*P* < 0.05) pH value and contained a higher (*P* < 0.05) level of available P and a lower (*P* < 0.05) level of available K than those at Kunming and Yuanjiang (Table 2). Apart from pH, organic C, and available P and K, the rhizosphere soils of *A. adenophora* from the different locations did not differ significantly (*P* > 0.05) in any other parameters.

**Table 2:** Physiochemical characterization of rhizosphere soils of *Ageratina adenophora* at different floristic regions of Kunming, Yunlong and Yuanjiang of Yunnan Province, China.

Soil sample site	pH	Organic C (g kg^−1^)	NH_4_^+^-N (mg kg^−1^)	NO_3_^—^-N (mg kg^−1^)	Total N (%)	Available P (mg kg^−1^)	Available K (mg kg^−1^)
Kunming	5.56 (±0.34)*	49.51 (±3.20)	2.54 (±0.22)	3.89 (±0.25)	1.07 (±0.14)	0.12 (±0.02)	0.21 (±0.03)
Yuanjiang	5.66 (±0.10)	14.10 (±1.12)	2.32 (±0.10)	4.40 (±0.15)	1.12 (±0.08)	0.13 (±0.10)	0.22 (±0.02)
Yunlong	7.43 (±0.38)	16.60 (±1.32)	2.62 (±0.12)	4.27 (±0.10)	1.41 (±0.16)	0.16 (±0.01)	0.14 (±0.01)

**Note:**

* Data before paratheses are the means of three replicate soil samples from each site, and data in the paratheses represent standard errors of the means.

### Screening for PGP bacteria from rhizosphere soils of *A. adenophora* from different floristic areas

**Figure 2:**
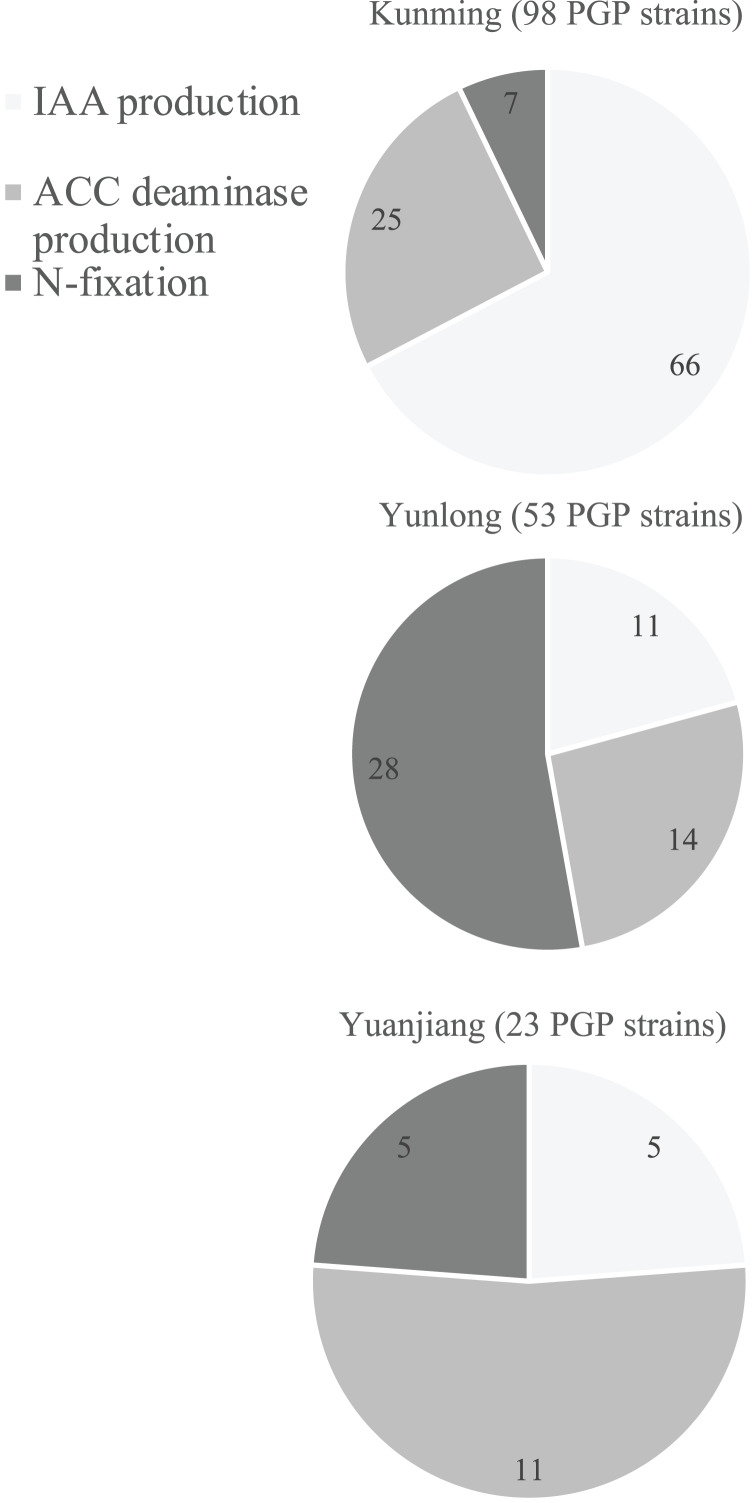
Diagram of the distribution of plant growth-promoting (PGP) bacterial strains isolated from the rhizosphere soil of *Ageratina adenophora* at Kunming (KM), Yunlong (YL) and Yuanjiang (YJ) of Yunnan Province, China. PGP bacteria indicate bacteria with *in vivo* nitrogen-fixation, indole-3-acetic acid (IAA) and 1-amino-cyclopropane-1-carboxylate (ACC) deaminase production ability.

Qualitative analyses using the selective media described above showed that 144 bacterial strains (Table S1) had at least one of the *in vitro* abilities for N-fixation, and IAA and ACC deaminase production (Table S1). To further identify those with the best PGP potentials we quantitatively analyzed each of their PGP abilities (Table S1). Soils from the different floristic areas differed in the numbers and composition of their PGP bacteria (Fig. 2). Of the 400 isolates from each study site, the rhizosphere soil from Kunming contained the most PGP bacteria (98 strains) followed by the soil samples from Yunlong (53 strains) and Yuanjiang (23 strains) (Fig. 2). Of the three PGP traits examined at each site, the IAA-producing bacteria were the most abundant (66 strains) in the soil at Kunming, while the N-fixing bacteria (28 strains) and the ACC deaminase-producing bacteria (11 strains) were the most abundant in the soils at Yunlong and Yuanjiang, respectively (Fig. 2).

### Phylogenetic composition and distribution of the PGP bacteria at the rhizosphere soils of *A. adenophora* at different floristic areas

**Figure 3:**
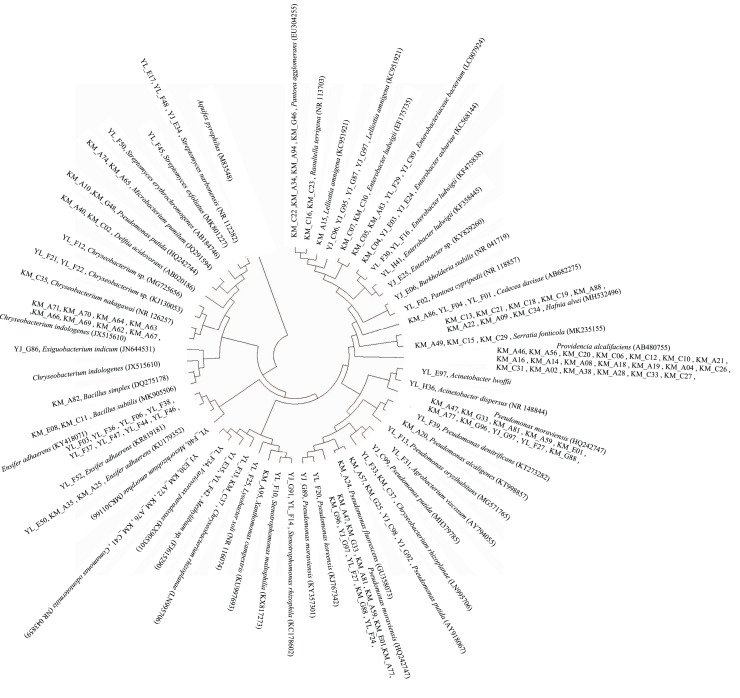
Diagram of a neighbor-joining tree showing phylogeny of the plant growth-promoting bacterial strains isolated from the rhizosphere soil of *Ageratina adenophora*. “KM”, “YL” and “YJ” represent Kunming, Yunlong and Yuanjiang of Yunnan Province, China, respectively.

The 16S rRNA gene sequence data revealed that a majority (62.5%) of the PGP bacteria identified were bacteria that were closely related (≥97.6% 16S rRNA gene sequence similarity) to members of the genera *Pseudomonas* (27 strains), *Providencia* (20 strains), *Chryseobacterium* (14 strains), *Ensifer* (12 strains), *Enterobacter* (nine strains), and *Hafnia* (eight strains) (Fig. 3, Table S1). Their abundances at the different study sites also differed. A total of six, five, and 15 *Pseudomonas* strains were found at Kunming, Yunlong, and Yuanjiang, respectively, while three *Enterobacteria* were recovered from each of the sites. A total of 10 and four *Chryseobacterium* strains, and two and 10 *Ensifer* strains, respectively, were recovered from the study sites of Kunming and Yunlong. *Providencia* (20) and *Hafnia* (eight) strains were found only at the Kunming site (Table S1).

### Correlation of invasion history of *A. adenophora* to the diversity and abundance of its rhizosphere PGP bacteria

The genus number and strain number of the PGP bacteria isolated from the rhizosphere soil of *A. adenophora* corresponded to the invasion history of *A. adenophora*. Thus, the study site of Kunming with a 10–12 years’ invasion history had the largest number (98 strains) and the most diverse (at least 19 genera) PGP bacteria, followed by that of Yunlong (65 strains, 15 genera) which had a 6–8 years’ invasion history. Yuanjiang had the fewest and least diverse bacteria (23 strains, 10 genera) with a short 2–3 years’ invasion history. Correlation analyses showed that significant positive correlations exist between the invasion years of *A. adenophora* and the genus number (*P* = 0.019) and the strain number *(P* = 0.022) of the rhizosphere PGP bacteria.

### Effects of the PGP bacterial strains on seed germination of crops

**Figure 4:**
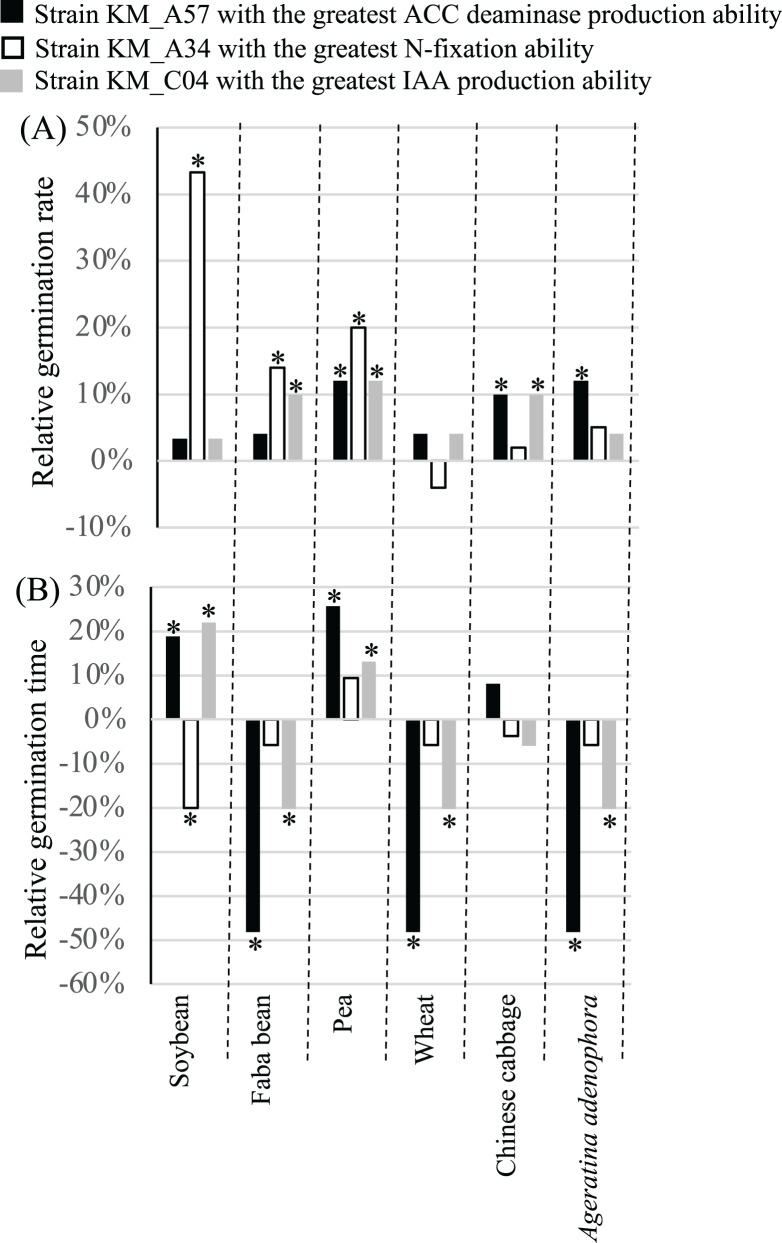
Relative germination rate (A) and germination time (B) of crops and *Ageratina adenophora* after seed inoculation with different PGP bacterial strains. ACC, 1-amino-cyclopropane-1-carboxylate; IAA, indole-3-acetic acid; an asterisk (*) indicates statistical significance (*P* < 0.05).

PGP strains KM_A34 of *Pantoea agglomerans* with the highest relative *nifH* gene expression level (1.1028), KM_C04 of *Enterobacter asburiae* with the highest IAA production ability of (35.02 mg L^−1^), and KM_A57 of *Pseudomonas putida* with the highest ACC deaminase production ability (9.23 U mg^−1^) (Table S1) were chosen. Their influence on the germination and growth of soybean, faba bean, pea, wheat, and Chinese cabbage, as well as *A. adenophora* was evaluated. Seed inoculation with the three PGP bacterial strains had different effects on the GR and GT of the crop seeds examined. Treatment with the N-fixation strain, KM_A34, significantly (*P* < 0.05) increased the GR of the soybean, faba bean, and pea seeds (Fig. 4A), while the application of the high ACC deaminase-producing strain, KM_A57, significantly (*P* < 0.05) increased the GR of the pea, Chinese cabbage, and *A. adenophora*. Treatment with the IAA-producing strain, KM_C04, significantly (*P* < 0.05) increased the GR of the faba bean, pea, and Chinese cabbage, while the N-fixation strain, KM_A34, significantly (*P* < 0.05) reduced the GT of soybean seeds (Fig. 4B). The application of the IAA-producing strain, KM_C04, significantly (*P* < 0.05) reduced the GT of the faba bean, wheat, and *A. adenophora* seeds, and increased that of the soybean and pea (Fig. 4B). The application of the ACC deaminase-producing strain, KM_A57, significantly (*P* < 0.05) reduced the GT of the faba bean, wheat, and *A. adenophora* seeds, and increased the GT of the soybean and pea seeds (Fig. 4B).

### Effects of the PGP bacterial strains on crop growth

**Figure 5:**
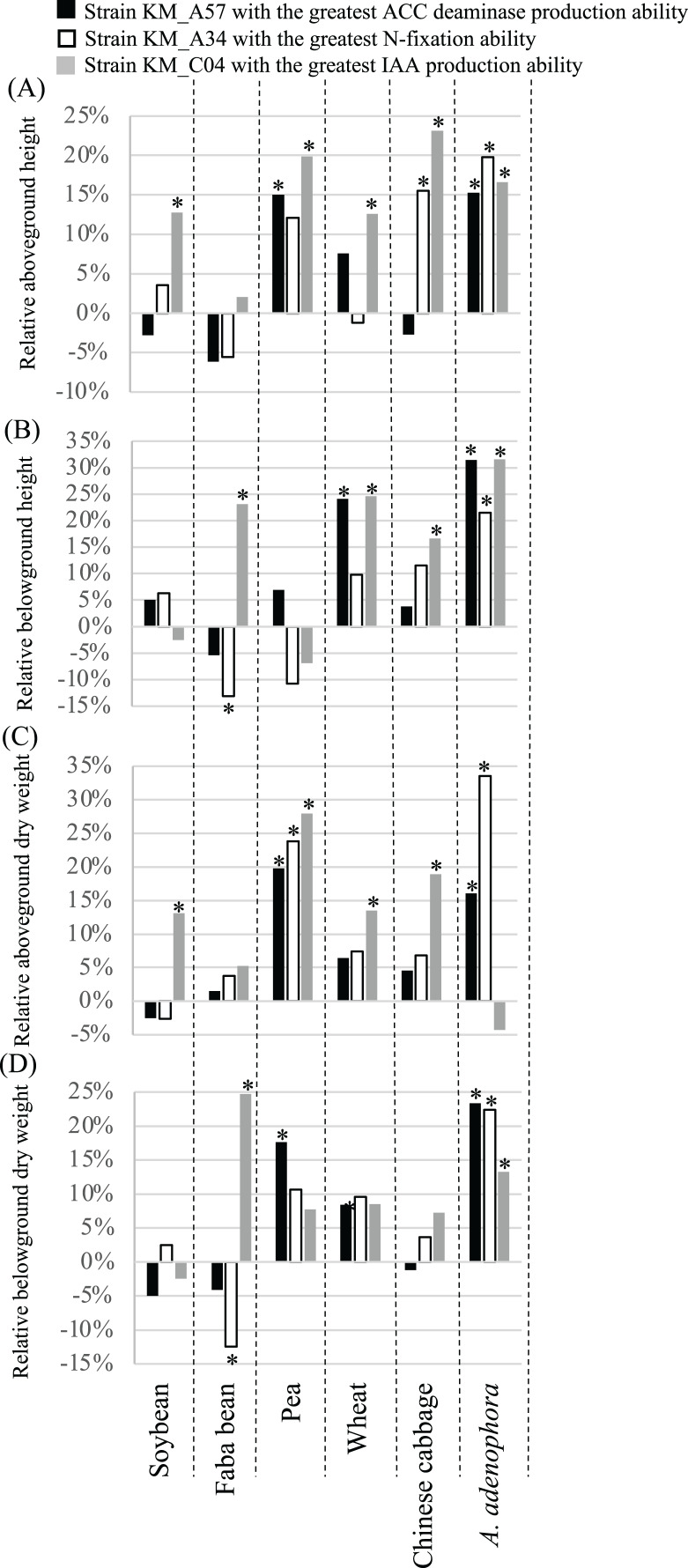
Relative aboveground height (A), belowground height (B), aboveground dry weight (C) and belowground dry weight (D) of crops and *Ageratina adenophora* after seed bacterization and root dipping with different PGP bacterial strains. ACC, 1-amino-cyclopropane-1-carboxylate; IAA, indole-3-acetic acid; an asterisk (*) indicates statistical significance (*P* < 0.05).

The 60-day greenhouse pot experiments were used to evaluate the effects of seed bacterization and root dip inoculation with the three PGP bacterial strains on the growth of the same crops examined above (Fig. 5). In comparison with the controls, the ACC deaminase-producing strain, KM_A57, significantly (*P* < 0.05) increased the crop’s aboveground height (pea) (Fig. 5A) and dry weight (pea) (Fig. 5C), and the belowground height (wheat) (Fig. 5B) and dry weight (pea) (Fig. 5D). The N-fixation strain, KM_A34, significantly (*P* < 0.05) increased the crops’ aboveground height (pea and Chinese cabbage) (Fig. 5A) and dry weight (pea) (Fig. 5C), and their belowground height (Chinese cabbage) (Fig. 5B) and dry weight (pea) (Fig. 5C). The strain also decreased the belowground height (Fig. 5B) and dry weight (Fig. 5D) of the faba bean. The IAA-producing strain, KM_C04, significantly (*P* < 0.05) increased the crops’ aboveground height (soybean, pea, wheat, Chinese cabbage) (Fig. 5A), dry weight (soybean, pea, wheat, Chinese cabbage) (Fig. 5C), belowground height (faba bean, wheat, Chinese cabbage) (Fig. 5B), and dry weight (faba bean) (Fig. 5D). All three PGP bacterial strains had a significant (*P* < 0.05), positive effect on the aboveground height (Fig. 5A) and dry weight (except for a neutral effect with the IAA-producing strain) (Fig. 5C), the underground height (Fig. 5B) and the dry weight (Fig. 5D) of *A. adenophora*.

## Discussion

We successfully achieved a comprehensive screening of selected PGP bacteria from the rhizosphere soils of *A. adenophora* for their growth-promoting abilities. The rhizosphere microbiota contains a diverse PGP bacterial community consisting of members of the phyla *Proteobacteria* (24 genera, 35 species, 118 strains), *Firmicutes* (three genera, four species, five strains), *Actinobacteria* (two genera, four species, seven strains), and *Bacteroidetes* (one genus, three species, 14 strains) (Table S1). The proteobacterial PGP members were the most abundant, comprising 80% genera, 76.1% species, and 81.9% strains. The number of PGP bacterial genera (30 genera) cultured in our study (Table S1) was more than that (22 genera) isolated by Xu et al. (2012) from the rhizosphere soils of *A. adenophora* existing in different areas of Yunnan, China. Such a difference may reflect the fact that they only screened PGP bacteria for their N-fixation ability, while here we used several metabolic features (N-fixation, IAA, and ACC deaminase production) and sampled soils exposed to different climatic conditions with different physiochemical characteristics. However, at the phylum level, the phylogenetic composition of PGP bacteria found in our study (Table S1) agrees closely with certain aspects of their data; both showed the *A. adenophora* PGP bacteria contained members of the *Proteobacteria, Firmicutes, Actinobacteria* and *Bacteroidetes* with members of the *Proteobacteria* being the most abundant.

Plants select and enrich the bacterial community composition of the rhizosphere by the composition of their root exudates. These exudates contain energy-rich carbon compounds and leaked photosynthates, including sugars, amino acids, and organic acids (Badri et al., 2009). Their composition and the rhizosphere community is unique to each plant species (Berg & Smalla, 2009). In addition to sugars, amino acids, and organic acids, root exudates and the associated plant litter of *A. adenophora* contain many antibacterial chemicals (Zhang et al., 2013). *A. adenophora* can alter the structure of its rhizosphere microbiota so that its competitive behaviour is enhanced (Wan et al., 2010), thus ensuring its successful establishment and dominance in native habitats (Yu et al., 2014). We found that diversity and abundance of the PGP bacteria correlated positively to the invasion history of *A. adenophora*. We fully noticed that in addition to a possible time-dependent enrichment of rhizosphere PGP bacteria by *A. adenophora*, these differences in diversity and abundance of the rhizosphere PGP bacteria may be due to the variations in climatic conditions and soil physiochemical properties of different floristic aeras.

The three PGP bacterial strains with the highest levels of N-fixation, IAA, and ACC deaminase production, respectively, had parameter-specific and crop-dependent effects on germination and growth parameters of the plants screened (Figs. 4 and 5, Table S1). For example, the N-fixation strain, KM_A34, promoted the GR of the soybean, faba bean, and pea seeds (Fig. 4A) but had a limited influence on all other crop growth-related parameters (Fig. 5). However, the ACC deaminase-producing strain, KM_A57, improved the GT (Fig. 4B) and different growth parameters of the pea, wheat, and faba bean (Fig. 5) but had little PGP effect on the those of Chinese cabbage (Figs. 4 and 5). The IAA-producing strain had a wide-spectrum effect by improving at least one of the germination- and growth-related parameters of each of the individual crops examined (Figs. 4 and 5). In particular, it improved the aboveground height (23.1%) and dry weight (18.9%) of the Chinese cabbage over controls (Fig. 5). The differences in the effects of these three PGP bacterial strains on different crops may reflect the differences in their competitive abilities *in situ* in being able to utilize the root exudates, soil physiochemical characteristics and composition, and the metabolic activity of the rhizosphere microbiota (Gupta et al., 2015).

PGPR may adopt a variety of mechanisms to influence plant growth. Indole acetic acid (IAA) is a common product of L-tryptophan metabolism produced by some bacteria, including PGPR, and is one of the most physiologically active auxins (Mohite, 2013). Improvement of crop growth results from seed inoculation with IAA-producing bacteria has been well-documented (Mohite, 2013; Kiruthika & Arunkumar, 2020; Batista et al., 2021). ACC deaminase produced by PGPR can also promote plant growth. Ethylene is a plant hormone that functions as an efficient plant growth regulator and plays an important role in normal development in plants as well as for their response to stress (Glick, 2005). Increased ethylene levels in plants exposed to various types of stress resulted in increased damage to the plant (Hyodo, 1991). ACC deaminase can reduce the immediate precursor of ethylene ACC to ammonia and α-ketobutyrate, thus reduce the harmful effects of ethylene on plant growth (Jackson, 1991). Seed inoculation with ACC deaminase producing bacteria improved crop growth under the limited availability of irrigation water (Gupta & Pandey, 2019; Zarei et al., 2020) and salt stress (Bal, Nayak & Adhya, 2013; Gowtham et al., 2020).

Free-living N-fixation by heterotrophic bacteria living on or near root surfaces is a significant source of N in some terrestrial systems. This is also of interest in crop production as an alternative to chemical fertilizer, potentially reducing production costs and ameliorating negative environmental impacts of fertilizer N additions (Igiehon & Babalola, 2018). In this study we found that inoculation with the N-fixation *Pantoea agglomerans* strain, KM_A34, improved the growth parameters of pea but not faba bean and soybean, which are all legumes that can carry out symbiotic N fixation with *Rhizobium*. Sibpondrung et al. (2020) found that the co-inoculation of *Bacillus* and *Bradyrhizobium* strains promoted nodule growth and the N-fixation of soybean. Similarly, Leite et al. (2022) reported that the co-inoculation of *Rhizobium* with *Bradyrhizobium* improved the productivity of common beans. Our result is a further confirmation to the results of these studies. Therefore, these helper bacteria cannot only enhance the effectiveness of the *Rhizobial* spp. but also improve crop productivity (Igiehon & Babalola, 2018). Furthermore, Remans et al. (2008) found a genotype-dependent effect of *Rhizobium-Azospirillum* coinoculation on the N-fixation and yield of two contrasting *Phaseolus vulgaris*. Their *Rhizobium-Azospirillum* co-inoculation had a positive effect on one of the bean genotype but a negative effect on the other. The results of our study in determining the difference in response of the pea, faba bean, and soybean to the treatment by the N-fixation strain, KM_A34, generally agrees with their results.

We found that the three PGP bacterial strains examined promoted the growth of the *A. adenophora* host more than the tested crops (Fig. 5). Of the four growth parameters measured (aboveground and belowground heights, and dry weights) for each plant, N-fixing KM_A34 and the ACC deaminase-producing KM_A57 had a significant (*P* < 0.05), positive influence on all of these parameters with *A. adenophora*. However, N-fixing KM_A34 and the ACC deaminase-producing KM_A57 did so on only less than one parameter (average 0.8) for each individual crops (Fig. 5). Fang et al. (2019) also reported that PGP bacteria isolated from the *A. adenophora*’s rhizosphere had host-specific, growth-promoting effects on seed germination timing, and aboveground and belowground growth of *A. adenophora* compared with two native species, *Fallopia multiflora* and *Arthraxon lanceolatus*. Of the 131 PGP isolates, 20.8% and 16.7% were beneficial for the aboveground and belowground growth of *A. adenophora*, respectively, but none were beneficial to the two native species. None of the PGP isolates negatively affected the performance of *A. adenophora*, but more than half of them showed detrimental effects on the belowground growth of the two native species. Of the five crops and six germination- and growth-parameters examined in our study, only the N-fixing KM_A34 and the ACC deaminase-producing KM_A57 had a negative effect on the GT of the pea (Fig. 4B), with a lower negative influence ratio than they reported for the native none-crop species.

## Conclusions

We found that a diverse PGP bacterial community with *in vitro* abilities of N-fixation, and the production of IAA and ACC deaminase, colonized the rhizosphere of the invasive *A. adenophora*. They are mostly members of phyla Proteobacteria, and the remaining are members of *Firmicutes*, *Actinobacteria*, and *Bacteroidetes*. Their diversity and relative abundance seem to correlate to the invasion history of the study sites. The IAA-producing strain, KM_C04, the ACC deaminase-producing strain, KM_A57, and N-fixation strain, KM_A34, increased significantly the GR and reduced the GT of the soybean, faba bean wheat, and/or pea. They promoted the aboveground and belowground heights and dry weights of the soybean, faba bean, pea and wheat, to varying degrees. The PGPR strains can be potentially used as bioinoculants to improve the growth and productivity of the soybean, faba bean, wheat, and pea.

## Supplemental Information

10.7717/peerj.15064/supp-1 Supplemental Information 1 Bacteria with *in vitro* plant growth-promoting attributes isolated from the rhizosphere soil of weed *Ageratina adenophora*at different floristic regions Kunming (KM), Yunlong (YL) and Yuanjiang (YJ) of Yunnan Province, China. ^a^ IAA: indole-3-acetic acid; ACC: 1-amino-cyclopropane-1-carboxylate. ^b^ Each value represents the mean relative *nifH* gene expressionlevel of individual bacterial strain (triplicate measurements) with a standard deviation of 3-5%. ^c^ – represents the plant growth-promoting attribute is undetectable.

10.7717/peerj.15064/supp-2 Supplemental Information 2 Growth related data of five crops and weed *Ageratina adenophora*.. Each data point indicates data of a growth-related parameter of a plant collected at day 60.

## Additional Information and Declarations

### DNA Deposition

The following information was supplied regarding the deposition of DNA sequences: All partial 16S rRNA sequence data of the PGP isolates have been deposited in the NCBI GenBank Nucleotide database under submission SUB8200347 *via* accession numbers MW037267 to MW037812.

### Data Availability

The following information was supplied regarding data availability: The raw measurements are available in the Supplemental Files.
