# Supplementary material for: Screening plant growth-promoting bacteria from the rhizosphere of invasive weed Ageratina adenophora for crop growth
Source: PeerJ. 2023 Mar 10;11:e15064. doi: 10.7717/peerj.15064 (PMC10010174; doi:10.7717/peerj.15064)
Supplement: Supplemental Information 1 — a IAA: indole-3-acetic acid; ACC: 1-amino-cyclopropane-1-carboxylate. b Each value represents the mean relative nifH gene expressionlevel of individual bacterial strain (triplicate measurements) with a standard deviation of 3-5%. c – represents the plant growth-promoting attribute is undetectable. [file peerj-11-15064-s001.pdf]

No.	Strains	Closely related bacterial species (strain No.) [similarity of 16S rRNA gene sequence]	Plant growth-promoting traits[a]		
			IAA production （mg L^{-1}）	ACC deaminase production（U mg^{-1}）	N-fixation （relative *nifH* gene expression level[b]）
1	YL_H36	*Acinetobacter dispersus* (NR 148844) [0.9979]	0.35 (±0.05)	–[c]	–
2	YL_E97	*Acinetobacter lwoffii* (AB859068) [0.9980]	–	–	1.0210
3	YL_F31	*Agrobacterium viscosum* (AY794055) [0.9986]	18.63 (±3.12)	–	–
4	KM_A82	*Bacillus simplex* (DQ275178) [0.9970]	10.58 (±2.18)	0.18 (±0.01)	1.0406
5	KM_E08	*Bacillus subtilis* (MK905506) [1]	–	0.39 (±0.01)	–
6	KM_C11	*Bacillus subtilis* (MK905506) [1]	–	0.02 (±0.00)	–
7	YJ_E06	*Burkholderia stabilis* (NR 041719) [0.9930]	–	–	1.0102
8	KM_A86	*Cedecea davisae* (AB682275) [0.9972]	14.78 (±2.12)	0.21 (±0.01)	–
9	YL_F04	*Cedecea davisae* (AB682275) [0.9972]	12.18 (±1.18)	3.17 (±0.02)	1.0310
10	YL_F01	*Cedecea davisae* (AB682275) [0.9972]	–	2.91 (±0.01)	1.0622
11	KM_A71	*Chryseobacterium indologenes* (JX515610) [0.9881]	15.37 (±3.07)	–	–
12	KM_A70	*Chryseobacterium indologenes* (JX515610) [0.9881]	14.68 (±3.45)	–	–
13	KM_A64	*Chryseobacterium indologenes* (JX515610) [0.9881]	12.23 (±2.12)	–	–
14	KM_A63	*Chryseobacterium indologenes* (JX515610) [0.9881]	11.68 (±2.01)	–	–
15	KM_A66	*Chryseobacterium indologenes* (JX515610) [0.9881]	10.5 (±1.18)	–	–
16	KM_A69	*Chryseobacterium indologenes* (JX515610) [0.9881]	10.03 (±1.67)	–	–
17	KM_A62	*Chryseobacterium indologenes* (JX515610) [0.9881]	8.82 (±1.34)	–	–
18	KM_A67	*Chryseobacterium indologenes* (JX515610) [0.9881]	7.58 (±1.02)	–	–
19	KM_C35	*Chryseobacterium nakagawai* (NR 126257) [0.9922]	0.78 (±0.12)	–	–
20	YL_F33	*Chryseobacterium rhizoplanae* (LN995706) [0.9901]	21.67 (±5.56)	–	–
21	KM_C37	*Chryseobacterium rhizoplanae* (LN995706) [0.9901]	1.95 (±0.07)	–	–
22	YL_F21	*Chryseobacterium* sp. (KJ130053) [0.9780]	–	0.09 (±0.01)	1.0208
23	YL_F22	*Chryseobacterium* sp. (KJ130053) [0.9760]	–	0.03 (±0.01)	–
24	YL_F12	*Chryseobacterium* sp. (MG725656) [0.9760]	11.50 (±1.07)	–	–
25	YJ_E30	*Comamonas odontotermitis* (NR 043859) [0.9986]	11.48 (±2.56)	–	–
26	KM_A72	*Comamonas odontotermitis* (NR 043859) [0.9986]	11.20 (±1.87)	–	–
27	KM_A76	*Comamonas odontotermitis* (NR 043859) [0.9986]	9.28 (±1.07)	–	–
28	KM_C41	*Comamonas odontotermitis* (NR 043859) [0.9986]	0.75 (±0.07)	–	–
29	KM_A40	*Delftia acidovorans* (AB020186) [0.9986]	16.88 (±4.46)	–	–
30	KM_C02	*Delftia acidovorans* (AB020186) [0.9986]	5.17 (±1.51)	–	–
31	YL_F52	*Ensifer adhaerens* (KR819181) [1]	–	0.41 (±0.02)	1.0704
32	YL_E50	*Ensifer adhaerens* (KU179352) [0.9986]	17.67 (±3.66)	–	–
33	KM_A35	*Ensifer adhaerens* (KU179352) [0.9986]	0.57 (±0.02)	–	–

34	KM_A25	*Ensifer adhaerens* (KU179352) [0.9986]	0.13 (±0.01)	–	–
35	YL_F03	*Ensifer adhaerens* (KY418071) [0.9980]	–	–	1.0304
36	YL_F36	*Ensifer adhaerens* (KY418071) [0.9980]	–	–	1.0406
37	YL_F06	*Ensifer adhaerens* (KY418071) [0.9980]	–	–	1.0426
38	YL_F38	*Ensifer adhaerens* (KY418071) [0.9980]	–	–	1.0301
39	YL_F37	*Ensifer adhaerens* (KY418071) [0.9980]	–	–	1.0402
40	YL_F47	*Ensifer adhaerens* (KY418071) [0.9980]	–	–	1.0428
41	YL_F44	*Ensifer adhaerens* (KY418071) [0.998]	–	–	1.0512
42	YL_F46	*Ensifer adhaerens* (KY418071) [0.998]	–	–	1.0398
43	KM_C04	*Enterobacter asburiae* (KC568144) [0.9966]	35.02 (±8.18)	1.90 (±0.02)	1.0422
44	YJ_E03	*Enterobacter asburiae* (KC568144) [0.9966]	21.98 (±4.73)	0.03 (±0.01)	–
45	YJ_E24	*Enterobacter asburiae* (KC568144) [0.9966]	2.82 (±0.41)	–	–
46	KM_C07	*Enterobacter ludwigii* (EF175735) [0.9979]	11.53 (±2.07)	–	–
47	KM_C30	*Enterobacter ludwigii* (EF175735) [0.9979]	0.90 (±0.01)	–	–
48	YL_H41	*Enterobacter ludwigii* (KF358445) [0.9952]	–	0.36 (±0.01)	–
49	YL_F30	*Enterobacter ludwigii* (KF475838) [0.9979]	20.43 (±3.92)	–	–
50	YL_F16	*Enterobacter ludwigii* (KF475838) [0.9979]	–	0.06 (±0.01)	1.0188
51	YJ_E25	*Enterobacter* sp. (KY829260) [0.9966]	21.78 (±3.87)	–	–
52	KM_C05	*Enterobacteriaceae bacterium* (LC007924) [0.9986]	15.63 (±2.57)	–	–
53	KM_A83	*Enterobacteriaceae bacterium* (LC007924) [0.9986]	–	0.82 (±0.01)	1.0176
54	YL_F29	*Enterobacteriaceae bacterium* (LC007924) [0.9986]	–	0.02 (±0.01)	1.0374
55	YJ_C89	*Enterobacteriaceae bacterium* (LC007924) [0.9986]	–	–	1.0266
56	YJ_G86	*Exiguobacterium indicum* (JN644531) [0.9980]	–	0.32 (±0.00)	–
57	KM_C13	*Hafnia alvei* (MH532496) [1]	33.00 (±5.69)	–	–
58	KM_C21	*Hafnia alvei* (MH532496) [1]	29.70 (±2.73)	0.21 (±0.01)	1.0408
59	KM_C18	*Hafnia alvei* (MH532496) [1]	17.97 (±1.66)	–	–
60	KM_C19	*Hafnia alvei* (MH532496) [1]	16.63 (±1.93)	–	–
61	KM_A88	*Hafnia alvei* (MH532496) [1]	15.92 (±1.77)	–	–
62	KM_A22	*Hafnia alvei* (MH532496) [1]	1.12 (±0.09)	–	–
63	KM_A09	*Hafnia alvei* (MH532496) [1]	0.62 (±0.01)	–	–
64	KM_C34	*Hafnia alvei* (MH532496) [0.9986]	0.58 (±0.01)	–	–
65	KM_A15	*Lelliottia amnigena* (KC951921) [0.9993]	–	0.59 (±0.01)	–
66	YJ_C96	*Lelliottia aquatilis* (MG916969) [0.9979]	–	0.57 (±0.01)	1.0141
67	YJ_G95	*Lelliottia aquatilis* (MG916969) [0.9979]	–	0.18 (±0.00)	–
68	YJ_G87	*Lelliottia aquatilis* (MG916969) [0.9980]	–	0.06 (±0.01)	–
69	YJ_G97	*Lelliottia aquatilis* (MG916969) [0.9979]	–	0.06 (±0.01)	–
70	YL_F25	*Lysobacter soli* (NR 116074) [0.9970]	–	0.22 (±0.01)	1.0509
71	YL_F40	*Mesorhizobium amorphae* (MK301166) [0.9956]	14.85 (±2.02)	–	–
72	YJ_E35	*Methylibium* sp. (FJ615290) [0.9890]	–	–	1.0632

73	YL_F42	*Methylibium* sp. (FJ615290) [0.9890]	–	–	1.0276
74	KM_A74	*Microbacterium pumilum* (JQ291594) [0.9903]	15.40 (±3.13)	–	–
75	KM_A65	*Microbacterium pumilum* (JQ291594) [0.9903]	10.65 (±1.56)	–	–
76	KM_C22	*Pantoea agglomerans* (EU304255) [0.9966]	28.90 (±2.45)	0.45 (±0.01)	–
77	KM_A34	*Pantoea agglomerans* (EU304255) [0.9966]	9.83 (±1.65)	0.47 (±0.01)	1.1028
78	KM_A94	*Pantoea agglomerans* (EU304255) [0.9966]	–	0.33 (±0.01)	–
79	KM_G46	*Pantoea agglomerans* (EU304255) [0.9966]	–	0.19 (±0.01)	–
80	YL_F02	*Pantoea cypripedii* (NR 118857) [0.9883]	12.05 (±2.12)	7.93 (±0.02)	1.0523
81	KM_A46	*Providencia alcalifaciens* (AB480755) [0.9959]	13.65 (±1.42)	–	–
82	KM_A56	*Providencia alcalifaciens* (AB480755) [0.9959]	13.52 (±1.33)	–	–
83	KM_C20	*Providencia alcalifaciens* (AB480755) (0.9959)	13.00 (±1.21)	–	–
84	KM_C06	*Providencia alcalifaciens* (AB480755) [0.9959]	12.58 (±1.09)	–	–
85	KM_C12	*Providencia alcalifaciens* (AB480755) [0.9959]	12.12 (±1.07)	–	–
86	KM_C10	*Providencia alcalifaciens* (AB480755) [0.9959]	10.60 (±1.05)	–	–
87	KM_A21	*Providencia alcalifaciens* (AB480755) [0.9959]	1.85 (±0.02)	–	–
88	KM_A16	*Providencia alcalifaciens* (AB480755) [0.9959]	1.80 (±0.02)	–	–
89	KM_A14	*Providencia alcalifaciens* (AB480755) [0.9959]	1.65 (±0.01)	–	–
90	KM_A08	*Providencia alcalifaciens* (AB480755) [0.9959]	1.62 (±0.01)	–	–
91	KM_A18	*Providencia alcalifaciens* (AB480755) [0.9959]	1.53 (±0.01)	–	–
92	KM_A19	*Providencia alcalifaciens* (AB480755) [0.9959]	1.53 (±0.01)	–	–
93	KM_A04	*Providencia alcalifaciens* (AB480755) [0.9959]	1.37 (±0.01)	–	–
94	KM_C26	*Providencia alcalifaciens* (AB480755) [0.9959]	1.33 (±0.01)	–	–
95	KM_C31	*Providencia alcalifaciens* (AB480755) [0.9959]	1.25 (±0.01)	–	–
96	KM_A02	*Providencia alcalifaciens* (AB480755) [0.9959]	1.23 (±0.01)	–	–
97	KM_A38	*Providencia alcalifaciens* (AB480755) [0.9959]	1.20 (±0.01)	–	–
98	KM_A28	*Providencia alcalifaciens* (AB480755) [0.9959]	1.15 (±0.01)	–	–
99	KM_C33	*Providencia alcalifaciens* (AB480755) [0.9959]	1.03 (±0.01)	–	–
100	KM_C27	*Providencia alcalifaciens* (AB480755) [0.9959]	0.97 (±0.01)	–	–
101	KM_A20	*Pseudomonas alcaligenes* (KT998857) [0.9952]	1.62 (±0.01)	–	–
102	YL_F39	*Pseudomonas denitrificans* (KT273282) [0.9980]	–	–	1.0233
103	KM_A24	*Pseudomonas fluorescens* (GU358073) [0.9979]	0.78 (±0.01)	–	–
104	YL_F20	*Pseudomonas koreensis* (KJ767342) [0.9979]	17.20 (±3.03)	0.43(±0.01)	
105	KM_A47	*Pseudomonas moraviensis* (HQ242747) [0.9972]	0.73 (±0.01)	–	–
106	KM_G33	*Pseudomonas moraviensis* (HQ242747) [0.9972]	–	1.87 (±0.01)	–
107	KM_A81	*Pseudomonas moraviensis* (HQ242747) [0.9972]	–	0.92 (±0.01)	–
108	KM_A59	*Pseudomonas moraviensis* (HQ242747) [0.9972]	–	0.61 (±0.01)	–
109	KM_E01	*Pseudomonas moraviensis* (HQ242747) [0.9972]	–	0.50 (±0.01)	–
110	KM_A77	*Pseudomonas moraviensis* (HQ242747) [0.9972]	–	0.39 (±0.01)	–
111	KM_G96	*Pseudomonas moraviensis* (HQ242747) [0.9972]	–	0.28 (±0.01)	–

112	YJ_G97	*Pseudomonas moraviensis* (HQ242747) [0.9979]	–	0.22 (±0.01)	–
113	YL_F27	*Pseudomonas moraviensis* (HQ242747) [0.9972]	–	0.11 (±0.01)	–
114	KM_G88	*Pseudomonas moraviensis* (HQ242747) [0.9972]	–	0.11 (±0.01)	–
115	YL_F24	*Pseudomonas moraviensis* (HQ242747) [0.9972]	–	0.04 (±0.01)	–
116	YJ_G89	*Pseudomonas moraviensis* (KY357301) [0.9979]	–	0.88 (±0.01)	–
117	KM_A79	*Pseudomonas mosselii* (LN995691) [0.9993]	–	0.14 (±0.01)	–
118	YL_F09	*Pseudomonas mosselii* (LN995691) [0.9993]	–	–	1.0175
119	YL_F13	*Pseudomonas oryzihabitans* (MG571765) [0.9993]	17.10 (±3.45)	–	1.0197
120	KM_A57	*Pseudomonas putida* (AY918067) [0.9979]	–	9.23 (±0.02)	1.0345
121	KM_G25	*Pseudomonas putida* (AY918067) [0.9979]	14.07 (±2.77)	8.36 (±0.02)	1 (as control)
122	YJ_C98	*Pseudomonas putida* (AY918067) [0.9979]	–	2.16 (±0.01)	–
123	YJ_G92	*Pseudomonas putida* (AY918067) [0.9979]	–	0.26 (±0.01)	–
124	KM_G62	*Pseudomonas putida* (AY918067) [0.9979]	–	0.04 (±0.01)	–
125	KM_A10	*Pseudomonas putida* (HQ242744) [0.9959]	0.83 (±0.01)	–	–
126	KM_G48	*Pseudomonas putida* (HQ242744) [0.9972]	–	3.20 (±0.01)	–
127	YJ_C99	*Pseudomonas putida* (MH379785) [1]	6.40 (±1.31)	–	–
128	KM_C16	*Raoultella terrigena* (NR 113703) [0.9945]	20.82 (±4.22)	–	–
129	KM_C23	*Raoultella terrigena* (NR 113703) [0.9945]	12.07 (±1.67)	–	–
130	KM_A49	*Serratia fonticola* (MK235155) [0.9979]	20.30 (±4.55)	–	–
131	KM_C15	*Serratia fonticola* (MK235155) [0.9979]	14.30 (±2.68)	–	–
132	KM_C29	*Serratia fonticola* (MK235155) [0.9979]	0.77 (±0.01)	–	–
134	KM_C39	*Staphylococcus epidermidis* (MK883070) [1]	1.45 (±0.01)	–	–
135	YL_F10	*Stenotrophomonas maltophilia* (KX817273) [0.9979]	–	1.00 (±0.01)	1.0221
136	YJ_G91	*Stenotrophomonas rhizophila* (KC178602) [0.9993]	–	0.03 (±0.01)	–
137	YL_F14	*Stenotrophomonas rhizophila* (KC178602) [0.9993]	–	–	1.0304
138	YL_F50	*Streptomyces erythrochromogenes* (AB184746) [0.999]	–	–	1.0420
139	YL_F45	*Streptomyces exfoliatus* (MK801227) [0.9979]	–	–	1.0423
140	YL_E17	*Streptomyces narbonensis* (NR 112282) [0.9970]	–	–	1.0231
141	YL_F48	*Streptomyces narbonensis* (NR 112282) [0.9970]	–	–	1.0234
142	YJ_E34	*Streptomyces narbonensis* (NR 112282) [0.9970]	–	–	1.0311
143	YL_F34	*Variovorax paradoxus* (KX905301) [0.9993]	–	0.35 (±0.01)	1.0821
144	KM_A95	*Xanthomonas campestris* (KU997693) [0.9993]	–	0.45 (±0.01)	–
